# Axial Length to Corneal Radius of Curvature Ratio (AL/CR) and Refractive Errors in a Single Center Romanian Population

**DOI:** 10.3390/biomedicines13112742

**Published:** 2025-11-10

**Authors:** Maria-Cristina Marinescu, Dana-Margareta-Cornelia Dascalescu, Dan Stanila, Sanda Jurja, Mihaela-Monica Constantin, Valeria Coviltir, Cristina Alexandrescu, Radu-Constantin Ciuluvica, Miruna-Gabriela Burcel

**Affiliations:** 1 Department of Medical Physiology, Faculty of Medicine, Carol Davila University of Medicine and Pharmacy, 050474 Bucharest, Romania; 2 Department of Ophthalmology, Faculty of Dentistry, Carol Davila University of Medicine and Pharmacy, 041292 Bucharest, Romania; 3 Department of Ophthalmology, Clinical Institute for Ophthalmological Emergencies “Prof. Dr. Mircea Olteanu”, 010464 Bucharest, Romania; 4 Department of Ophthalmology, Faculty of Medicine, Lucian Blaga University, 550169 Sibiu, Romania; 5 Department of Ophthalmology, Faculty of Medicine, Ovidius University, 900470 Constanta, Romania; jurjasanda@yahoo.com; 6 Department of Ophthalmology, Oftaclinic, 030987 Bucharest, Romania; 7 Department of Ophthalmology, Faculty of Medicine, Carol Davila University of Medicine and Pharmacy, 050474 Bucharest, Romania; 8 Department of Anatomy, Faculty of Dentistry, Carol Davila University of Medicine and Pharmacy, 050474 Bucharest, Romania; 9 Faculty of Medicine, Transilvania University of Braşov, 500019 Braşov, Romania; miruna.burcel@unitbv.ro; 10 Brasov County Emergency Clinical Hospital, 500326 Braşov, Romania

**Keywords:** myopia, hyperopia, axial length, corneal radius of curvature, corneal hysteresis, corneal resistance factor

## Abstract

**Background**: Refractive errors are a common ophthalmological complaint, with a significant potential on the quality of life of our patients—myopia in particular has a growing incidence worldwide. Recent research focused on the ratio between the axial length of the eye (AL) and the corneal radius of curvature (CR), as it had proven valuable in refractive error diagnosis, and risk of progression and of complications. The objective of the study is to compare young emmetropic, hyperopic, and myopic eyes in terms of corneal biomechanics and ocular biometry, focusing on the AL/CR ratio. **Methods**: This cross-sectional study included 144 myopic eyes, 92 emmetropic eyes, and 47 hyperopic eyes. Measurements included cycloplegic autorefractometry (SE—spherical equivalent), Ocular Response Analyzer (CH—corneal hysteresis, CRF—corneal resistance factor), Aladdin biometry (AL, CR, ACD—anterior chamber depth, CCT—central corneal thickness, AL/CR ratio). **Results**: ACD, AL, and AL/CR were significantly higher and CCT, SE, CH, and CRF were lower in myopia. The AL/CR ratio correlated positively with AL and ACD and negatively with SE and CR in myopes and hyperopes, and correlated positively with AL and negatively with SE, CH, CRF, and CCT in emmetropes. **Conclusions**: The AL/CR ratio is significantly higher in myopes and significantly lower in hyperopes, compared to emmetropes, with differences also being in biomechanical properties (CH, CRF) and morphological ones (AL, CCT, ACD). This suggests the AL/CR ratio as a future potential biomarker for refractive errors, particularly for their risk of progression and complications.

## 1. Introduction

Refractive errors represent an emerging ophthalmological domain, with a growing understanding of their importance and impact on vision. Firstly, myopia is the refractive error with the most rapidly increasing prevalence. The World Health Organisation warns that 52% of the world’s population will be myopic by 2050 [1,2]. In the European region, the current prevalence, in the 2020–2030 decade, is estimated to vary from 32.2% in Eastern Europe to 36.7% in Western Europe, with a value of 34.6% in Central Europe, to which Romania, in which the current study was performed, belongs [2]. Moreover, the prevalence varies greatly between European countries: a recent meta-analysis reveals the lower prevalence of 11.9% in Finland and 49.7% in Sweden. In terms of age groups, the highest prevalence is in adolescents (25.2%), followed by adults (24.3%), and lowest in children between 6 and 11 years old, at 5.5% [3]. Data in Asian countries point to an even bigger problem—myopia is diagnosed in 91% of first year students in Taiwan universities [4], and in 96% of army recruits in South Korea [5], with over 20% of these cases being high myopia [4,5].

High myopia is a known risk factor for several ophthalmic pathologies, such as myopic macular degeneration and retinal detachment. Even a small refractive error increases the risk: an eye with a refractive error of −3.00 diopters (D) has a three times higher risk of retinal detachment compared with a −1.00 D refractive error [6]. Myopic macular degeneration is the main cause of blindness among myopic patients [7,8]. Another chronic disease associated with myopia is primary open angle glaucoma (POAG), the risk of developing POAG being 2.26 times higher compared to the general population [9].

Another significant refractive error, followed in the present study, is hyperopia, which is associated with a high risk of lowered quality of life [10]. Prevalence worldwide is 4.6% in children and 30.9% in adults, with a high degree of variation between geographical regions [11]. Uncorrected hyperopia, particularly anisometropia (difference of more than one diopter of spherical equivalent between eyes [12]) raises the risk of amblyopia in children (defined as a decreased visual acuity, in the absence of organic ocular disease, caused by sensory deprivation throughout the development of the eye, most commonly caused by uncorrected strabismus and refractive errors [13]). This hyperopia–amblyopia association was identified in a pediatric population in Romania as well [12]. Uncorrected amblyopia brings also an altered perception of personal wellbeing, particularly in young people, but also an altered perception of general and mental health [14]. In terms of visual adverse effects, hyperopia raises the risk for adult patients to develop primary angle closure glaucoma (PACG)—a spherical equivalent of 1–3 diopters involves a 58% higher risk of PACG, with values over 3 diopters involving a 3.33 times higher risk, and even more in young patients under 65 years old [15].

In the process of myopic progression, the axial length axis increases, and a flattening of the cornea has been described in the literature as a compensatory phenomenon in some cases [16], a change described since 1988 by Grosvenor [17]. As an imbalance between axial elongation and corneal compensation occurs, the actual refractive error, myopia, is developed, which has also been described as a mismatch between the refractive power of the ocular dioptric apparatus (cornea, lens) and the elongated axial length [16,18]. In the recent literature, the ratio between axial length (in millimeters) and corneal curvature radius (in millimeters) (AL/CR) is intensely studied [19]. A high AL/CR ratio was also associated with another important myopia risk factor: the parental myopia [19]. The interaction between axial elongation and corneal flattening may also give us clues about future complications of myopia—in a study that included over 1000 children with low myopia (SE between −3.00 D and −0.50 D), there was observed an association between a flatter cornea, a larger axial length, and a higher frequency and severity of the initial form of myopic maculopathy, with fundus tessellation [20].

Not only do biometric ocular data offer us insight into the risk of the above-mentioned complications, but also biomechanical properties of the cornea, which have previously been proven valuable in pathology, such as glaucoma or keratoconus [21,22], and also in the subject of our research, hyperopia and myopia [23,24]. As such, our hypothesis is that, in the present Romanian population of young myopes and hyperopes, the AL/CR ratio will correlate both with ocular biometrics and corneal biomechanics. The objective of the present study is to study the morphological ocular data in relation to the biomechanical properties of the cornea, namely the corneal hysteresis and resistance factor, in both myopic and hyperopic eyes.

## 2. Methods

This study was performed using a non-randomized cross-sectional methodology. The study group was constituted by evaluating inclusion and exclusion criteria regarding all patients who presented to a private ophthalmology clinic in Bucharest, Romania, in the period February–June 2023. The study was conducted in accordance with the Declaration of Helsinki and approved by the Carol Davila University of Medicine and Pharmacy Research Ethics Committee (protocol code PO-35-F-03/16.01.2023). All patients (and their legal guardians for patients under the age of 18) offered informed consent.

The inclusion criterion was the spherical equivalent (SE) (spherical refractive error + 1/2 cylindrical refractive error), measured after pharmacological cycloplegia (cyclopentolate or tropicamide, instilled 3 times at 5 min intervals in each eye). The myopic group had SE lower than −0.50 D [25], hyperopic group had SE over 0.50 D [26], and emmetropia represented an SE in the ±0.50 D range [27], also acting as a control group.

Patients were excluded if they presented any other ocular pathology, other than the refractive errors mentioned above (hyperopia and myopia). They were excluded if they presented presbyopia, corneal disease such as keratoconus, amblyopia, strabismus, glaucoma or ocular hypertension, cataract, retinal disease, or a history of refractive surgery. Also, systemic diseases such as diabetes mellitus, autoimmune disorders, arterial hypertension, and dyslipidemia constituted contraindications, together with pregnancy and breastfeeding.

A complete ophthalmological examination was performed for all patients, including visual acuity testing using the Snellen chart, a complete slit-lamp exam, intraocular pressure measurement using Goldmann tonometer, and autorefractometry after pharmacological cycloplegia, using the Topcon KR800 device (Topcon, Tokyo, Japan) to measure the spherical equivalent by directing infrared light into the eye and measuring the reflection of the light from the retina [28].

The patients also underwent a series of measurements: corneal biomechanical properties were measured using the Ocular Response Analyzer (ORA) (Reichert Ophthalmic Instruments Inc., Depew, NY, USA), which results in corneal hysteresis (CH), corneal resistance factor (CRF), and the Goldmann-correlated intraocular pressure IOP (IOPg). ORA is a non-contact tonometer, using infrared light to detect the deformation of the cornea, which is caused by an air puff emitted by the device. The cornea becomes applanated and then concave, before resuming the initial shape. The device records the pressures corresponding to the two applanation moments and calculates the intraocular pressure of two parameters of the corneal response to the mechanical deformation: CH, representing the corneal capacity to absorb and dissipate energy (the difference between the two applanations), and CRF, reflecting the corneal resistance and elasticity (similar to CH, the second applanation multiplied with a constant) [29].

Ocular biometry was performed using the Aladdin biometer (Topcon, Tokyo, Japan), which utilizes low-coherence light [30] and was used to measure the axial length (AL), average corneal curvature radius (CR), anterior chamber depth (ACD), and central corneal thickness (CCT). The AL/CR ratio was calculated as follows:

$$AL/CR=\frac{Axial\;length\;of\;the\;eye\;(AL\;-measured\;in\;millimetres)}{Corneal\;curvature\;radius\;(CR\;-\;measured\;in\;millimetres)}$$

### Statistical Analysis

The right eye of every patient was randomly chosen to be included in the analysis. Only quality measurements were included (for instance, Waveform score over 7 for the ORA reading). Statistical analysis of the data was performed using the statistical package IBM SPSS Statistics for Windows, version 26 (IBM Corp., Armonk, NY, USA).

This study includes both categorical and numerical, continuous data. The absolute and relative frequencies were calculated for categorical data. For numerical data, the average and standard deviation (SD) were determined. The age was classified into several age groups: 6–10 years old, 11–14 years old, 15–18 years old, and over 18 years old. The normal distribution of the numeric variables was verified and confirmed using the Kolmogorov–Smirnov Test.

To find significant differences between any two groups (hyperopic and emmetropic, myopic and emmetropic, male and female, children and adults), an independent samples t-test was used, preceded by the Levene’s Test for Equality of variances (as a homogenous variance of the means in the two compared samples is an assumption of the independent samples *t*-test) [31]. In order to find significant differences between more than two groups (age brackets 6–10 years old, 11–14 years old, 15–18 years old, and over 18 years old), the one-way ANOVA analysis was performed, followed by the post hoc Bonferroni analysis.

The degree of correlation between variables was ascertained by computing Pearson’s correlation coefficient, known as “Pearson’s r”, which can vary between 1 and −1. A moderate correlation is between 0.3 and 0.5 or between −0.3 and −0.5, a strong correlation is over 0.5 or below −0.5, and a weak correlation is between 0.3 and −0.3, according to Pearson’s r. A *p*-value of 0.05 is regarded as the cutoff point for statistical significance.

## 3. Results

### 3.1. Descriptive Results

In the entire study group, composed of 283 patients, a female predominance is observed (60.4%, *n* = 171) compared to the male gender (39.6%, *n* = 112) (see Table 1). In the myopic group (M), out of the total of 144 participants, 59 (41.0%) are male and 85 (59.0%) are female. In the group of emmetropes (E), the distribution is similar, with 37 (40.2%) male participants and 55 (59.8%) female, out of a total of 92 participants. In regard to the hypermetropia group (H), out of the total of 47 participants, 16 (34.0%) are male and 31 (66.0%) are female. The average of all followed variables, broken down on refractive group, age group, and gender can be found in Supplementary Material Table S1.

The average age in the entire cohort was 20.23 (SD 9.21), with the youngest patients being hyperopic, with a mean of 17.81 (SD 11.58), the mean age in the myopia group being 19.66 (SD 7.38), and emmetropes being oldest at 22.35 (SD 10.08). The distribution into the age ranges of 6–10 years, 11–14 years, 15–18 years, and >18 years is illustrated in Table 2.

Several significant differences were also detected between men and women. Women had significantly lower CCT (555 μm vs. 569, *p* = 0.001), ACD (3.63 mm vs. 3.77 mm, *p* = 0.001), AL (23.75 mm vs. 24.36, *p* < 0.001), and CR (7.68 mm vs. 7.82 mm, *p* < 0.001) (see Table 3)—i.e., thinner.

### 3.2. Differences Between Refractive Groups

**Figure 1:**
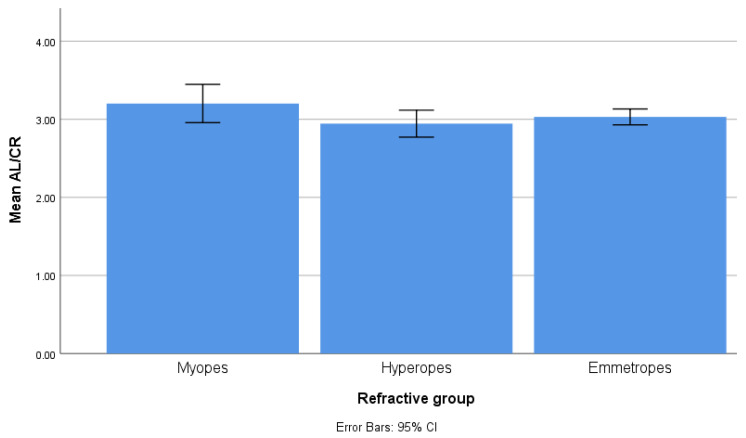
Bar chart illustrating the average AL/CR ratio in the myopic (3.20), emmetropic (3.03), and hyperopic groups (2.94), the value being significantly different between the three groups. The bars represent the upper and lower limits of the 95% confidence intervals of the values.

**Figure 2:**
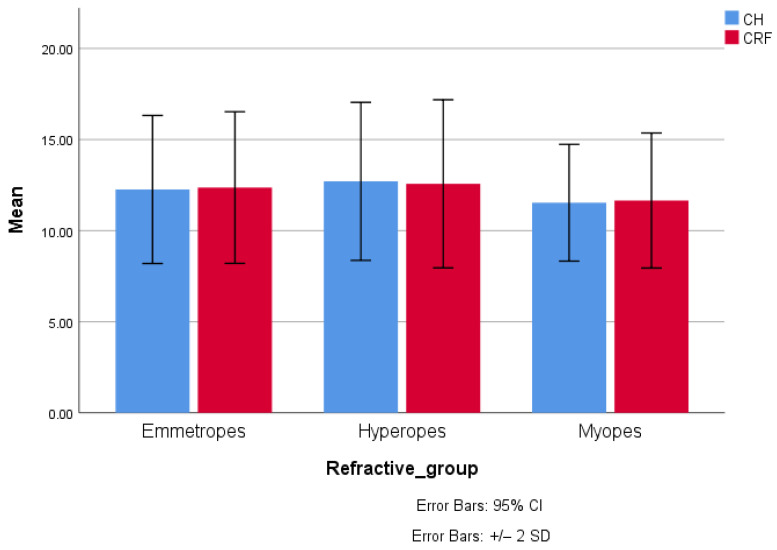
Bar charts illustrating the average CH (blue bar) and CRF (red bar) in myopia, emmetropia, and hyperopia. Values are significantly lower in myopes, while there are no differences between emmetropes and hyperopes. The bars represent the upper and lower limits of the 95% confidence intervals of the values.

There were several significant differences between the three refractive error groups (see Table 4). As SE also constitutes the inclusion criteria, it was significantly lower in myopia and higher in hyperopia. ACD, AL, and AL/CR were significantly higher in myopia and lower in hyperopia, compared to emmetropia (Figure 1). CCT, CH, and CRF were significantly lower in myopic eyes, without differences between emmetropic and hyperopic eyes (Figure 2).

### 3.3. Differences Between Age Groups

In order to follow the above-mentioned variables over critical periods of refractive development, they were compared over the different age ranges. In the myopic group, the ANOVA test revealed statistically significant differences between age ranges in **CH** (differed between the 11–14 years and >18 years groups and between the 11–14 years and 15–18 years groups), **CRF** (between the 11–14 years and >18 years groups), **ACD** (between the 11–14 years and >18 years groups)—see Table 5A,B.

In the emmetropia group, the ANOVA test revealed age-related differences in **SE** (between 15–18 years and 6–10 years and between 15–18 years and >18 years), **CH** (between 6–10 years and >18 years and between 15–18 years and >18 years), **ACD** (between 11–14 years and >18 years and between 15–18 years and >18 years), **AL** (between 6–10 years and 11–14 years), **CR** (between 11–14 years and 15–18 years and between 11–14 years and >18 years) and **AL/CR** (between 6–10 years and all other age groups)—see Table 5A,B.

Lastly, hyperopes presented significant differences in **CCT** (between 11–14 years and 15–18 years) and **ACD** (between 6–10 years and >18 years)—see Table 5A,B.

### 3.4. Correlations Between AL/CR and Other Variables

The focus of this study is the relevance of the AL/CR ratio, and the wide correlations with other ocular variables constitute an argument for its usage in clinical practice. In the myopic group, AL/CR had a strong positive correlation with AL but a weak negative correlation with CR, a strong negative correlation with SE (stronger than the AL–SE correlation), and a weak positive correlation with ACD (similar to the AL–ACD correlation). CR has positive correlations with AL (strong correlation) and CCT (weak correlation) (see Table 6).

The AL/CR ratio also correlated well in the emmetropic group (see Table 7). The correlation with AL and with SE was weak, and there was no correlation with CR. In addition to the myopic group, there were weak–moderate correlations with CH, CRF, and CCT. AL had a moderate positive correlation with ACD, and CR had a strong positive correlation with AL, and weak positive correlations with CCT and ACD.

Lastly, AL/CR has moderate–strong correlations with AL and CR in the hyperopic group (Table 8). It had a strong negative correlation with SE (stronger than the AL–SE correlation). ACD had a moderate positive correlation both with AL/CR and with AL, and AL–CR was a strong positive correlation.

### 3.5. Multiple Linear Regression Analysis of Analyzed Variables

Lastly, a multiple linear regression analysis was performed, in order to describe the relationship among all following variables. As the three subgroups, particularly hyperopes, represent smaller samples, and as the number of variables was higher, the analysis was performed on the entire cohort (Table 9). As we can see, 86.3% of the SE variation in our analyzed data can be explained by the variation in AL/CR, CRF, Kavg, ACD, CCT, CH, and AL, and this association is statistically significant (*p* value of regression model is <0.001). The statistically significant variables are ACD and the AL/CR ratio; a 1 mm change in ACD is expected to be associated with a change of 0.059 D of SE, and a change of 1 unit in the AL/CR ratio is expected to be associated with a change of 1.043 diopters of SE.

## 4. Discussion

As far as we are aware, this is the first study investigating the AL/CR ratio in a Romanian population involving both adults and children and both myopic and hyperopic eyes. The strong connections found between this ratio and corneal biomechanical properties, ocular parameters, and particularly ones involved in the follow-up of refractive errors (axial length and spherical equivalent) make it a valuable biomarker for our patients. In the myopic group, corneal hysteresis (CH) and the corneal resistance factor (CRF) are significantly higher in adolescents (11–14 years) compared to adults (>18 years), suggesting a decrease in ocular biomechanical properties with age, an aspect correlated with the progression of myopia. Also, anterior chamber depth (ACD) is significantly greater in adolescents than in adults, which may reflect ocular morphological changes specific to growth and myopic progression. In emmetropes, a reduction in corneal biomechanical properties is observed in adults (>18 years) compared to younger groups. Also, axial length (AL) and AL/CR ratio increase during childhood, reaching a plateau in adolescence and adulthood. Lastly, in hypermetropes, higher CCT and ACD values are found in the pediatric and adolescent groups compared to adults. These variations suggest the involvement of anatomical factors in the maintenance of hypermetropia at young ages, with a tendency to reduce the depth of the anterior chamber with advancing age.

In our cohort, we note an average SE of −3.75 D in the young school aged myopes (6–10 years old), therefore suggesting that the process of myopization debuts at a very young age. Moreover, it is known that the rate of progression is greatest in the 6–10 age bracket [32], therefore supporting the need for screening for early myopia development in pre-school and primary school children.

Interestingly, the AL/CR ratio was significantly lower in younger children compared to adolescents and adults amongst emmetropes (2.96, versus values over 3). As the risk of SE progression in the 6–10 age group of emmetropes is high, and may evolve towards myopia [33], it is particularly important to follow young emmetropes who may develop subsequent myopia.

As expected, myopic eyes differed significantly in terms of ocular morphology, with lower SE and CCT and higher AL and ACD. Conversely, hyperopes had significantly higher SE and lower ACD and AL. The difference in CCT, detected only in myopes, has been previously described [34], and may have a direct relationship with SE—a large study detected a decrease in CCT of 1 micrometer for each 1.00 D of SE [35]. The difference in ACD reflects the connection between hyperopia and a shallow anterior chamber, with a higher risk of PACG [15,36]. Data from the literature suggest that the steepest decrease in ACD appears in the 20–30 age group (similar to our cohort) [37].

In terms of corneal biomechanics, the myopic group differed significantly, but the hyperopic one did not, compared to emmetropes, therefore suggesting a particular behavior of the myopic cornea. This characteristic of the myopic eye may prove a valuable biomarker for refractive progression, as research is starting to describe low CH as correlated to a greater AL increase in myopic children using single vision spectacles [38]. On the other hand, in children undergoing orthokeratology, the baseline CH did not associate with AL progression, suggesting a benefit in molding the cornea and, implicitly, altering the corneal biomechanics in young myopes [38]. Furthermore, other biomechanical properties, measured with the Corvis ST device, have correlated with AL progression: a high corneal amplitude of deformation (DA) correlates with faster myopic progression [39].

The importance of the AL/CR ratio has been previously studied: it was shown that AL/CR percentiles are a better predictor of myopia than AL percentiles, and a value over 3 can be used as a diagnostic criterion for myopia [40]. In accordance, the values in our study were 3.2 in myopes, 3.03 in emmetropes, and 2.94 in hyperopes, with similar averages in other studies [41].

Similarly to other studies [19], the correlation with SE was stronger for the AL/CR ratio compared to the AL, in both refractive error groups. Interestingly, in emmetropes, SE did not correlate with either AL or CR, but it did correlate with the ratio between them, supporting this biomarker’s role in understanding the evolution of refractive errors. Interestingly, the correlation AL/CR–ACD was strongest in the hyperopic group, supporting the potential role of the ratio in the early detection of hyperopes at risk of acute angle closure and PACG. However, as the study is cross-sectional and the cohort of hyperopes is the smallest of the three compared in the present study, more research is needed in order to draw a firm conclusion.

In our cohort, children and adults were significantly different. A decrease in CH and CRF has been described in the literature with aging, and may be related to the decrease in elasticity due to protein glycation and oxidative stress which contributes to physiological crosslinking [42], this correlation being significant even when taking into account gender, corneal thickness, intraocular pressure, and refractive error [43]. Further, a genome-wide association study has also found that CH and CRF decrease with age, identifying a difference between sexes which was absent in our cohort [44]. The difference in ACD may prove valuable, as advanced age is a known risk factor for angle closure glaucoma [36,45]. Also, adults are known to have thinner corneas, both in a Caucasian cohort [46] and in an Asian cohort [47]. As a theoretical consideration, beyond the scope of our study, this phenomenon of crosslinking has been investigated as a potential therapeutic option for progressive myopia [48]; however, it is not yet established as secure and efficacious as in other ocular diseases, primarily in keratoconus [49].

Male and female patients differed only in terms of morphological parameters, not in terms of the AL/CR ratio or the biomechanical properties: females have a lower CCT, ACD, and AL and a higher CR. However, there are conflicting results in the literature: large scale studies have either found no difference in AL and CCT [47,50], a difference only in CH and CRF, but not in CCT [51], an increase in CCT in men only in the 18–29 age group [46], or in all age groups in myopes [52,53]. However, gender has been described as an important factor in myopia: annual progression is faster in girls than in boys [54], differences being evident starting from the age of nine [55]. A steeper cornea has been previously described in females [52,53], including children—a study in which for the same refractive error, girls had a steeper cornea and shorter axial length [40]. This interesting observation has not been replicated in our cohort—while female patients had a lower AL and higher CR, AL/CR did not significantly differ—showing that there is more research needed to determine the role of refractive value and ethnicity on the AL/CR gender difference.

In tandem with the lower ACD in adult patients, women also present a lower ACD. Both represent risk factors for PACG [37,45], suggesting a need for closer follow-up of female patients, particularly hyperopic ones, as they age.

The study of refractive errors is of paramount importance, as their impact has been documented starting from young ages: children with uncorrected refractive errors have lower reading and writing test scores [56]. Public health measures and raising awareness have been proven to help—a study which involved the visual acuity correction of a cohort of children has led to improvements in testing scores [57].

In terms of myopia, several behavioral risk factors have been identified: long time spent performing near work (reading, writing, and using electronic devices up close) and short time spent outdoors [58] and alterations enhanced by the COVID-19 prevention measures [59], with reliable data showing accelerated myopic progression during isolation [60]. The development and progression of myopia involve several factors and are incompletely understood mechanisms [61]. In humans, it is currently believed that the mechanism for myopic progression is peripheral retinal hyperopic defocus. An image, if formed clearly on the fovea, in the periphery is projected posterior to the retina, and therefore a hyperopic defocus is created, which acts as a trigger for axial lengthening of the eye [62]. In animal models, the phenomenon of form deprivation myopia has also been proven important, in which the absence of normal light stimuli to the eye during its development leads to severe axial elongation and choroidal and scleral thinning. This proves that adequate light stimulation of the retina is involved in the normal pathway of eye growth [61,63]. In the relationship with the peripheral defocus described in humans, an important mechanism described in animal models is the induction of myopia by placing a divergent lens in front of the animal’s eye, with this hyperopic defocus leading to choroidal thinning, increased axial length, and, ultimately, a myopic refractive error [63].

The biochemical pathways triggered by peripheral defocus involve neurotransmitters such as dopamine and GABA, growth modulators such as retinoic acid and insulin, and also nitric oxide and melanopsin at the retinal level. The choroid and retinal pigmentary epithelium face changes in the blood flow, ion transport, and growth modulation (TGFβ, FGF) [61,64]. The scleral remodeling contributing to the axial elongation has been characterized by an increase in matrix metalloproteinases and a decrease in tissue inhibitors of metalloproteinases, and therefore a loss of collagen and proteoglycans [65]. This scleral thinning also involves the transformation of fibroblasts into myofibroblasts and an up-regulation of ischemic signaling cascades [66].

On the other hand, hyperopia involves a short axial length relative to the ocular dioptric power; therefore, the image does not form on the fovea, but behind it, particularly if the object is at a near distance to the eye [67]. During the early years of life, the eye undergoes the process of emmetropisation—the eye is hyperopic at birth and the refractive error decreases during childhood, mainly through axial lengthening, on average reaching emmetropia around the age of seven [61]. It is believed that persistent hyperopia is due to a disturbance in the normal process and persistence of initial positive refractive errors [68].

This study presents a series of limitations: as the study was performed in a single clinical center, it presents the risk of limited external validity, and more research in diverse populations is needed in order to generalize the findings herein. The cross-sectional design inhibits the ability to create time predictions, and the small hyperopic group reduces the statistical power for hyperopia-specific analyses. Most importantly, due to time constraints and the presentation of the patients to the clinic, the cohort is skewed, with a lower number of hyperopic eyes in the analysis.

## 5. Conclusions

The present study confirms that in the Romanian population as well, the AL/CR ratio is significantly higher in myopes (value over 3) and significantly lower in hyperopes (under 3). This suggests that the AL/CR ratio may act as a valuable biomarker for refractive errors, and may prove significant in the risk of myopic progression and complications.

## Figures and Tables

**Table 1:** Contingency table illustrating the distribution of participants according to gender and the refractive group to which they belong, namely myopes (M), emmetropes (E), and hypermetropes (H).

Crosstab			Gender		Total
			Male	Female	
Study group	M (myopic)	Count	59	85	144
		%	41.0%	59.0%	100%
	E (emmetropes)	Count	37	55	92
		%	40.2%	59.8%	100%
	H (hyperopes)	Count	16	31	47
		%	34.0%	66.0%	100%
Total		Count	112	171	283
		%	39.6%	60.4%	100%

**Table 2:** Contingency table illustrating the distribution of participants according to age group and the refractive group to which they belong, namely myopes (M), emmetropes (E), and hypermetropes (H).

*Crosstab*			Age				Total
			6–10	11–14	15–18	>18	
Study group	M (myopic)	Count	8	31	37	68	144
		%	5.6%	21.5%	25.7%	47.2%	100.0%
	E (emmetropes)	Count	9	20	17	46	92
		%	9.8%	21.7%	18.5%	50.0%	100.0%
	H (hyperopes)	Count	17	10	2	18	47
		%	36.2%	21.3%	4.3%	38.3%	100.0%
Total		Count	34	61	56	132	283
		%	12.0%	21.6%	19.8%	46.6%	100.0%

**Table 3:** Average values of variables which differ significantly between men and women in the entire cohort: central corneal thickness (CCT), anterior chamber depth (ACD), axial length (AL), corneal radius of curvature (CR).

	Sex	Mean	Std. Deviation	Mean Difference	Sig. (2-Tailed)
CCT (μm)	Female	555.0	38.0	−15.0	0.001
	Male	569.0	36.0		
ACD (mm)	Female	3.626	0.351	−0.139	0.001
	Male	3.765	0.300		
AL (mm)	Female	23.745	1.209	−0.611	<0.001
	Male	24.356	1.229		
CR (mm)	Female	7.677	0.252	−0.140	<0.001
	Male	7.816	0.265		

**Table 4:** Average values of variables in the myopic, emmetropic, and hyperopic group. * denotes statistical significance of t-test comparison to the emmetropia values (*p* value under 0.05): spherical equivalent (SE), corneal hysteresis (CH), corneal resistance factor (CRF), intraocular pressure Goldman-correlated (IOPG), central corneal thickness (CCT), anterior chamber depth (ACD), axial length (AL), corneal radius of curvature CR), AL/CR ratio.

Study Group		Mean	Std. Deviation	*p* Value	Mean Difference
M (myopic)	SE (D) *	−3.056	1.993	<0.001	−3.079
	CH (mmHg) *	11.532	1.602	0.002	−0.727
	CRF (mmHg) *	11.656	1.852	0.007	−0.710
	CCT (μm) *	554.0	38.0	0.008	−14.0
	ACD (mm) *	3.790	0.321	<0.001	0.170
	AL (mm) *	24.686	1.150	<0.001	0.447
	CR (mm)	7.712	0.267	0.064	−0.066
	AL/CR *	3.202	0.123	<0.001	0.172
E (emmetropes)	SE (D)	0.023	0.322		
	CH (mmHg)	12.259	2.031		
	CRF (mmHg)	12.365	2.080		
	CCT (μm)	568.0	38.0		
	ACD (mm)	3.621	0.273		
	AL (mm)	23.568	0.822		
	CR (mm)	7.779	0.267		
	AL/CR	3.030	0.051		
H (hyperopes)	SE (D) *	1.891	1.581	<0.001	1.867
	CH (mmHg)	12.704	2.168	0.234	0.446
	CRF (mmHg)	12.570	2.308	0.597	0.205
	CCT (μm)	566.0	35.0	0.845	−1.0
	ACD (mm) *	3.468	0.375	0.019	−0.153
	AL (mm) *	22.663	0.686	<0.001	−0.906
	CR (mm)	7.702	0.251	0.106	−0.076
	AL/CR *	2.944	0.086	<0.001	−0.086

**Table 5:** (**A**) ANOVA analysis results, revealing the parameters which are different in a statistically significant manner between age groups, in each refractive group. (**B**) Post hoc Bonferroni analysis results, revealing the specific age groups between which there are statistically significant differences, in each refractive group. All mean differences are significant at the 0.05 level.

**(A)**					
**Study Group**			**Mean Square**	**F**	***p* Value**
M (myopic)	CH (mmHg)		16.367	7.211	<0.001
	CRF (mmHg)		22.527	7.453	<0.001
	ACD (mm)		0.443	4.644	0.004
E (emmetropes)	SE (D)		0.413	4.433	0.006
	CH (mmHg)		26.843	8.007	<0.001
	CRF (mmHg)		14.957	3.774	0.013
	ACD (mm)		0.536	9.226	<0.001
	AL (mm)		2.798	4.641	0.005
	CR (mm)		0.237	3.611	0.016
	AL/CR		0.014	6.486	0.001
H (hyperopes)	CCT (μm)		0.004	3.550	0.022
	ACD (mm)		0.833	9.393	<0.001
(**B**)					
**Study Group**	**Dependent Variable**	**(I) Age**	**(J) Age**	**Mean Difference (I-J)**	***p* Value**
M (myopic)	CH (mmHg)	11–14	15–18	1.086	0.022
			>18	1.456	<0.001
		15–18	11–14	−1.086	0.022
		>18	11–14	−1.456	<0.001
	CRF (mmHg)	11–14	>18	1.726	<0.001
		>18	11–14	−1.726	<0.001
	ACD (mm)	11–14	>18	0.238	0.005
		>18	11–14	−0.238	0.005
E (emmetropes)	SE (D)	6–10	15–18	0.408	0.010
		15–18	6–10	−0.408	0.010
			>18	−0.266	0.017
		>18	15–18	0.266	0.017
	CH (mmHg)	6–10	>18	2.905	<0.001
		15–18	>18	1.490	0.031
		>18	6–10	−2.905	<0.001
			15–18	−1.490	0.031
	ACD (mm)	11–14	>18	0.267	0.001
		15–18	>18	0.311	<0.001
		>18	11–14	−0.267	0.001
			15–18	−0.311	<0.001
	AL (mm)	6–10	11–14	−1.057	0.006
		11–14	6–10	1.057	0.006
	CR (mm)	11–14	15–18	0.257	0.019
			>18	0.188	0.045
		15–18	11–14	−0.257	0.019
		>18	11–14	−0.188	0.045
	AL/CR	6–10	11–14	−0.066	0.004
			15–18	−0.077	0.001
			>18	−0.072	<0.001
		11–14	6–10	0.066	0.004
		15–18	6–10	0.077	0.001
		>18	6–10	0.072	<0.001
H (hyperopes)	CCT (μm)	11–14	15–18	−81.0	0.013
		15–18	11–14	8.01	0.013
	ACD (mm)	6–10	>18	0.431	0.001
		11–14	>18	0.567	<0.001
		>18	6–10	−0.431	0.001
			11–14	−0.567	<0.001

**Table 6:** Statistically significant correlations between variables followed in the study and the AL/CR ratio, with AL and CR taken separately, in the myopic group (sample size = 144). * reflects statistical significance of *p* value under 0.05, ** *p* value under 0.01.

		AL/CR	AL	CR
Pearson Correlation	**AL**	0.684 **		0.587 **
Sig. (2-tailed)		<0.001		<0.001
Pearson Correlation	**CR**	−0.189 *		
Sig. (2-tailed)		0.023		
Pearson Correlation	**SE**	−0.894 **	−0.632 **	
Sig. (2-tailed)		<0.001	<0.001	
Pearson Correlation	**CCT**			0.221 **
Sig. (2-tailed)				0.008
Pearson Correlation	**ACD**	0.272 **	0.314 **	
Sig. (2-tailed)		0.001	<0.001	

**Table 7:** Statistically significant correlations between variables followed in the study and the AL/CR ratio, with AL and CR taken separately, in the emmetropic group. * reflects statistical significance of *p* value under 0.05, ** *p* value under 0.01.

		AL/CR	AL	CR
Pearson Correlation	AL	0.276 **		0.887 **
Sig. (2-tailed)		0.008		<0.001
Pearson Correlation	SE	−0.298 **		
Sig. (2-tailed)		0.004		
Pearson Correlation	CH	−0.335 **		
Sig. (2-tailed)		0.001		
Pearson Correlation	CRF	−0.289 **		
Sig. (2-tailed)		0.005		
Pearson Correlation	CCT	−0.318 **		0.242 *
Sig. (2-tailed)		0.002		0.020
Pearson Correlation	ACD		0.323 **	0.226 *
Sig. (2-tailed)			0.002	0.035

**Table 8:** Statistically significant correlations between variables followed in the study and the AL/CR ratio, with AL and CR taken separately, in the hyperopic group. * reflects statistical significance of *p* value under 0.05, ** *p* value under 0.01.

		AL/CR	AL	CR
Pearson Correlation	AL	0.397 **		0.564 **
Sig. (2-tailed)		0.006		<0.001
Pearson Correlation	CR	−0.534 **		
Sig. (2-tailed)		<0.001		
Pearson Correlation	SE	−0.696 **	−0.560 **	
Sig. (2-tailed)		<0.001	<0.001	
Pearson Correlation	ACD	0.337 *	0.358 *	
Sig. (2-tailed)		0.025	0.017	

**Table 9:** Multiple linear regression model for the dependent variable: SE (spherical equivalent, in diopters), reporting unstandardized (B) and standardized (Beta) regression coefficients, along with each coefficient’s *p* value. At the end of the table can be found the *p* value of the likelihood of the model to be statistically significant. * marks the statistically significant variables in the equation.

**Model Summary for Dependent Variable: SE**							
R	R Square	Adjusted R Square		Std. Error of the Estimate			
0.929	0.863	0.859		0.96148			
Predictors: (Constant), AL/CR, CRF, KAVG, ACD, CCT, CH, AL							
**Model**	**Sum of Squares**	**df**	**Mean Square**	**F**	***p* value**		
Regression	1498.151	7	214.022	231.515	<0.001		
Residual	238.505	258	0.924				
Total	1736.656	265					
	**Unstandardized Coefficients**		**Standardized Coefficients**	**t**	***p* value**	**95.0% Confidence Interval for B**	
	**B**	**Std. Error**	**Beta**			**Lower Bound**	**Upper Bound**
(Constant)	39.525	27.090		1.459	0.146	−13.821	92.870
CH (mmHg)	−0.003	0.062	−0.003	−0.055	0.956	−0.126	0.120
CRF (mmHg)	−0.033	0.065	−0.026	−0.515	0.607	−0.161	0.094
CCT (micrometers)	0.783	2.190	0.012	0.357	0.721	−3.530	5.095
ACD (mm)	0.447	0.205	0.059	2.175	0.031 *	0.042	0.851
Kavg (D)	0.232	0.622	0.136	0.373	0.709	−0.993	1.458
AL (mm)	0.164	1.142	0.080	0.143	0.886	−2.086	2.413
AL/CR	−18.197	8.826	−1.043	−2.062	0.04 *	−35.577	−0.817

## Data Availability

The datasets generated and/or analyzed during the current study are available from the corresponding author on reasonable request.

## Supplementary Materials

The following supporting information can be downloaded at https://www.mdpi.com/article/10.3390/biomedicines13112742/s1, Table S1: Baseline characteristics table summarizing key demographic and ocular parameters of the study population, primary stratification by refractive error (myopia, emmetropia, hyperopia), secondary by age group and sex.

## Abbreviations

The following abbreviations are used in this manuscript:

ACD	Anterior chamber depth
AL	Axial length
CCT	Central corneal thickness
CH	Corneal hysteresis
CR	Corneal curvature radius
CRF	Corneal resistance factor
IOPg	Goldmann-correlated intraocular pressure
ORA	Ocular Response Analyzer
SE	Spherical equivalent

## References

[1] World Health Organization–Brien Holden Vision Institute (2015). The Impact of Myopia and High Myopia.

[2] Holden B.A., Fricke T.R., Wilson D.A., Jong M., Naidoo K.S., Sankaridurg P., Wong T.Y., Naduvilath T.J., Resnikoff S. (2016). Global Prevalence of Myopia and High Myopia and Temporal Trends from 2000 through 2050. Ophthalmology.

[3] Moreira-Rosário A., Lanca C., Grzybowski A. (2025). Prevalence of Myopia in Europe: A Systematic Review and Meta-Analysis of Data from 14 Countries. Lancet Reg. Health-Eur..

[4] Wang T.-J., Chiang T.-H., Wang T.-H., Lin L.L.-K., Shih Y.-F. (2009). Changes of the Ocular Refraction among Freshmen in National Taiwan University between 1988 and 2005. Eye.

[5] Jung S.-K., Lee J.H., Kakizaki H., Jee D. (2012). Prevalence of Myopia and Its Association with Body Stature and Educational Level in 19-Year-Old Male Conscripts in Seoul, South Korea. Investig. Ophthalmol. Vis. Sci..

[6] Cooper J., Tkatchenko A.V. (2018). A Review of Current Concepts of the Etiology and Treatment of Myopia. Eye Contact Lens.

[7] Holden B., Sankaridurg P., Smith E., Aller T., Jong M., He M. (2014). Myopia, an Underrated Global Challenge to Vision: Where the Current Data Takes Us on Myopia Control. Eye.

[8] Buch H., Vinding T., La Cour M., Appleyard M., Jensen G.B., Nielsen N.V. (2004). Prevalence and Causes of Visual Impairment and Blindness among 9980 Scandinavian Adults: The Copenhagen City Eye Study. Ophthalmology.

[9] Wu J., Hao J., Du Y., Cao K., Lin C., Sun R., Xie Y., Wang N. (2022). The Association between Myopia and Primary Open-Angle Glaucoma: A Systematic Review and Meta-Analysis. Ophthalmic Res..

[10] Kandel H., Khadka J., Goggin M., Pesudovs K. (2017). Impact of Refractive Error on Quality of Life: A Qualitative Study. Clin. Experiment. Ophthalmol..

[11] Hashemi H., Fotouhi A., Yekta A., Pakzad R., Ostadimoghaddam H., Khabazkhoob M. (2018). Global and Regional Estimates of Prevalence of Refractive Errors: Systematic Review and Meta-Analysis. J. Curr. Ophthalmol..

[12] Mocanu V., Horhat R. (2018). Prevalence and Risk Factors of Amblyopia among Refractive Errors in an Eastern European Population. Medicina.

[13] Xiang Z.-Y., Zou H.-D. (2020). Recent Epidemiology Study Data of Myopia. J. Ophthalmol..

[14] Bountziouka V., Cumberland P.M., Rahi J.S. (2021). Impact of Persisting Amblyopia on Socioeconomic, Health, and Well-Being Outcomes in Adult Life: Findings from the UK Biobank. Value Health.

[15] Shen L., Melles R.B., Metlapally R., Barcellos L., Schaefer C., Risch N., Herrinton L.J., Wildsoet C., Jorgenson E. (2016). The Association of Refractive Error with Glaucoma in a Multiethnic Population. Ophthalmology.

[16] Fan Y., Huang Y., Huang X. (2024). Association between Axial Length to Corneal Curvature Radius Ratio and Myopia in Adult Patients. J. Ophthalmol..

[17] Grosvenor T. (1988). High Axial Length/corneal Radius Ratio as a Risk Factor in the Development of Myopia. Am. J. Optom. Physiol. Opt..

[18] Muralidharan A.R., Lança C., Biswas S., Barathi V.A., Shermaine L.W.Y., Seang-Mei S., Milea D., Najjar R.P. (2021). Light and Myopia: From Epidemiological Studies to Neurobiological Mechanisms. Ther. Adv. Ophthalmol..

[19] Matsumura S., Dannoue K., Kawakami M., Uemura K., Kameyama A., Takei A., Hori Y. (2022). Prevalence of Myopia and Its Associated Factors Among Japanese Preschool Children. Front. Public Health.

[20] Gong W., Cheng T., Wang J., Zhang B., Chen J., Zhu J., Zou H., Liu K., He X., Xu X. (2023). Role of Corneal Radius of Curvature in Early Identification of Fundus Tessellation in Children with Low Myopia. Br. J. Ophthalmol..

[21] Potop V., Coviltir V., Corbu C., Burcel M.G., Ionescu C.I., Dascalescu D.M.C. (2019). Corneal Hysteresis, A Glaucoma Risk Factor Independent of the Intraocular Pressure. Rev. Roum. Sci. Techn.–Électrotechn. Énerg..

[22] Burcel M.G., Constantin M., Ionita G., Dascalescu D., Ionescu C., Stanila D., Potop V., Coviltir V. (2020). Levels of Lactoferrin, Lysozyme and Albumin in the Tear Film of Keratoconus Patients and Their Correlations with Important Parameters of the Disease. Rev. Romana Med. Lab..

[23] Marinescu M., Dascalescu D., Constantin M., Coviltir V., Burcel M., Darabus D., Ciuluvica R., Stanila D., Voinea L., Potop V. (2023). Corneal Biomechanical Properties in Myopic and Emmetropic Children. Eur. Rev. Med. Pharmacol. Sci..

[24] Marinescu M.-C., Dascalescu D.-M.-C., Constantin M.-M., Coviltir V., Potop V., Stanila D., Constantin F., Alexandrescu C., Ciuluvica R.-C., Voinea L.-M. (2023). Particular Anatomy of the Hyperopic Eye and Potential Clinical Implications. Medicina.

[25] Chuck R.S., Jacobs D.S., Lee J.K., Afshari N.A., Vitale S., Shen T.T., Keenan J.D. (2018). Refractive Errors & Refractive Surgery Preferred Practice Pattern^®^. Ophthalmology.

[26] Monika M., Durajczyk M. (2023). Evaluation of the Prevalence of Refractive Defects and Ocular Function in a Group of 1518 Children Aged 8 Years in Northwestern Poland-A Retrospective Study. J. Clin. Med. Res..

[27] Hagen L.A., Gilson S.J., Akram M.N., Baraas R.C. (2019). Emmetropia Is Maintained Despite Continued Eye Growth From 16 to 18 Years of Age. Investig. Ophthalmol. Vis. Sci..

[28] Brodie S.E. (2022). 2022–2023 Basic and Clinical Science Course, Section 03: Clinical Optics and Vision Rehabilitation Print.

[29] Roberts C.J., Liu J. (2017). Corneal Biomechanics: From Theory to Practice.

[30] Mandal P., Berrow E.J., Naroo S.A., Wolffsohn J.S., Uthoff D., Holland D., Shah S. (2014). Validity and Repeatability of the Aladdin Ocular Biometer. Br. J. Ophthalmol..

[31] Section 3.3: Independent T-Test Assumptions, Interpretation, and Write Up. https://usq.pressbooks.pub/statisticsforresearchstudents/chapter/independent-t-test-assumptions/.

[32] Lam C.S., Edwards M., Millodot M., Goh W.S. (1999). A 2-Year Longitudinal Study of Myopia Progression and Optical Component Changes among Hong Kong Schoolchildren. Optom. Vis. Sci. Off. Publ. Am. Acad. Optom..

[33] Kim Y.J., Kim T.G. (2023). Analysis of 2-Year Spherical Equivalent Progression in Emmetropic Children with Non-Cycloplegic Refraction: A Retrospective Chart Review. BMC Ophthalmol..

[34] Fam H.-B., How A.C.S., Baskaran M., Lim K.-L., Chan Y.-H., Aung T. (2006). Central Corneal Thickness and Its Relationship to Myopia in Chinese Adults. Br. J. Ophthalmol..

[35] Bradfield Y.S., Melia B.M., Repka M.X., Kaminski B.M., Davitt B.V., Johnson D.A., Kraker R.T., Manny R.E., Matta N.S., Pediatric Eye Disease Investigator Group (2011). Central Corneal Thickness in Children. Arch. Ophthalmol..

[36] Weinreb R.N., Aung T., Medeiros F.A. (2014). The Pathophysiology and Treatment of Glaucoma: A Review. JAMA.

[37] Phu J., Tong J., Zangerl B., Le J.L., Kalloniatis M. (2020). Cluster Analysis Reveals Patterns of Age-related Change in Anterior Chamber Depth for Gender and Ethnicity: Clinical Implications. Ophthalmic Physiol. Opt..

[38] Wan K., Cheung S.W., Wolffsohn J.S., Orr J.B., Cho P. (2018). Role of Corneal Biomechanical Properties in Predicting of Speed of Myopic Progression in Children Wearing Orthokeratology Lenses or Single-Vision Spectacles. BMJ Open Ophthalmol..

[39] Xiang K., Chen J., Zhao W., Zhu Z., Ding L., Bulloch G., Du L., Xu X., Zhu M., He X. (2023). Changes of Corneal Biomechanics in Children Using Orthokeratology and Their Roles in Predicting Axial Length Progression-A Prospective 2-Year Study. Acta Ophthalmol..

[40] He X., Sankaridurg P., Naduvilath T., Wang J., Xiong S., Weng R., Du L., Chen J., Zou H., Xu X. (2023). Normative Data and Percentile Curves for Axial Length and Axial Length/corneal Curvature in Chinese Children and Adolescents Aged 4–18 Years. Br. J. Ophthalmol..

[41] Iyamu E., Iyamu J., Obiakor C.I. (2011). The Role of Axial Length-Corneal Radius of Curvature Ratio in Refractive State Categorization in a Nigerian Population. ISRN Ophthalmol..

[42] Murphy M.L., Pokrovskaya O., Galligan M., O’Brien C. (2017). Corneal Hysteresis in Patients with Glaucoma-like Optic Discs, Ocular Hypertension and Glaucoma. BMC Ophthalmol..

[43] Sharifipour F., Panahi-bazaz M., Bidar R., Idani A., Cheraghian B. (2016). Age-Related Variations in Corneal Biomechanical Properties. J. Curr. Ophthalmol..

[44] Simcoe M.J., Khawaja A.P., Hysi P.G., Hammond C.J., UK Biobank Eye and Vision Consortium (2020). Genome-Wide Association Study of Corneal Biomechanical Properties Identifies over 200 Loci Providing Insight into the Genetic Etiology of Ocular Diseases. Hum. Mol. Genet..

[45] Zhang N., Wang J., Chen B., Li Y., Jiang B. (2020). Prevalence of Primary Angle Closure Glaucoma in the Last 20 Years: A Meta-Analysis and Systematic Review. Front. Med..

[46] Galgauskas S., Juodkaite G., Tutkuvienė J. (2014). Age-Related Changes in Central Corneal Thickness in Normal Eyes among the Adult Lithuanian Population. Clin. Interv. Aging.

[47] Li Y., Fu Z., Liu J., Li M., Zhang Y., Wu X. (2017). Corneal Endothelial Characteristics, Central Corneal Thickness, and Intraocular Pressure in a Population of Chinese Age-Related Cataract Patients. J. Ophthalmol..

[48] Yasir Z.H., Sharma R., Zakir S.M. (2023). Scleral Collagen Cross Linkage in Progressive Myopia. Indian J. Ophthalmol..

[49] Burcel M.G., Lacraru I.C., Dascalescu D.M.C., Corbu M.-C., Potop V., Coviltir V. (2022). Assessment of Two-Year Clinical Outcomes after Keratoconus Treatment Using Two Different Crosslinking Protocols. Eur. Rev. Med. Pharmacol. Sci..

[50] Hashmani N., Hashmani S., Murad A., Asghar N., Islam M. (2019). Effect of Demographic Variables on the Regional Corneal Pachymetry. Asia Pac. J. Ophthalmol..

[51] Strobbe E., Cellini M., Barbaresi U., Campos E.C. (2014). Influence of Age and Gender on Corneal Biomechanical Properties in a Healthy Italian Population. Cornea.

[52] Wang Q., Liu W., Wu Y., Ma Y., Zhao G. (2017). Central Corneal Thickness and Its Relationship to Ocular Parameters in Young Adult Myopic Eyes. Clin. Exp. Optom..

[53] Yang S., Jiang Y., Cui G., Li Y. (2022). Age- and Gender-Related Characteristics of Astigmatism in a Myopic Population. Front. Med..

[54] Jones-Jordan L.A., Sinnott L.T., Chu R.H., Cotter S.A., Kleinstein R.N., Manny R.E., Mutti D.O., Twelker D.J., Zadnik K. (2021). Myopia Progression as a Function of Sex, Age, and Ethnicity. Investig. Ophthalmol. Vis. Sci..

[55] Rudnicka A.R., Kapetanakis V.V., Wathern A.K., Logan N.S., Gilmartin B., Whincup P.H., Cook D.G., Owen C.G. (2016). Global Variations and Time Trends in the Prevalence of Childhood Myopia, a Systematic Review and Quantitative Meta-Analysis: Implications for Aetiology and Early Prevention. Br. J. Ophthalmol..

[56] Banashefski B., Rhee M.K., Lema G.M.C. (2023). High Myopia Prevalence across Racial Groups in the United States: A Systematic Scoping Review. J. Clin. Med..

[57] Neitzel A.J., Wolf B., Guo X., Shakarchi A.F., Madden N.A., Repka M.X., Friedman D.S., Collins M.E. (2021). Effect of a Randomized Interventional School-Based Vision Program on Academic Performance of Students in Grades 3 to 7: A Cluster Randomized Clinical Trial. JAMA Ophthalmol..

[58] Czepita M., Czepita D., Lubiński W. (2017). The Influence of Environmental Factors on the Prevalence of Myopia in Poland. J. Ophthalmol..

[59] Wang J., Li Y., Musch D.C., Wei N., Qi X., Ding G., Li X., Li J., Song L., Zhang Y. (2021). Progression of Myopia in School-Aged Children After COVID-19 Home Confinement. JAMA Ophthalmol..

[60] Picotti C., Sanchez V., Fernandez Irigaray L., Iurescia A., Iribarren R. (2022). Rapid Progression of Myopia at Onset during Home Confinement. J. Am. Assoc. Pediatr. Ophthalmol. Strabismus.

[61] Troilo D., Smith E.L., Nickla D.L., Ashby R., Tkatchenko A.V., Ostrin L.A., Gawne T.J., Pardue M.T., Summers J.A., Kee C.-S. (2019). IMI—Report on Experimental Models of Emmetropization and Myopia. Investig. Ophthalmol. Vis. Sci..

[62] Németh J., Tapasztó B., Aclimandos W.A., Kestelyn P., Jonas J.B., De Faber J.-T.H.N., Januleviciene I., Grzybowski A., Nagy Z.Z., Pärssinen O. (2021). Update and Guidance on Management of Myopia. European Society of Ophthalmology in Cooperation with International Myopia Institute. Eur. J. Ophthalmol..

[63] Chakraborty R., Ostrin L.A., Benavente-Perez A., Verkicharla P.K. (2020). Optical Mechanisms Regulating Emmetropisation and Refractive Errors: Evidence from Animal Models. Clin. Exp. Optom..

[64] Tedja M.S., Haarman A.E.G., Meester-Smoor M.A., Kaprio J., Mackey D.A., Guggenheim J.A., Hammond C.J., Verhoeven V.J.M., Klaver C.C.W., CREAM Consortium (2019). IMI—Myopia Genetics Report. Investig. Ophthalmol. Vis. Sci..

[65] Guo L., Frost M.R., He L., Siegwart J.T., Norton T.T. (2013). Gene Expression Signatures in Tree Shrew Sclera in Response to Three Myopiagenic Conditions. Investig. Ophthalmol. Vis. Sci..

[66] Zhou X., Zhang S., Zhang G., Chen Y., Lei Y., Xiang J., Xu R., Qu J., Zhou X. (2020). Increased Choroidal Blood Perfusion Can Inhibit Form Deprivation Myopia in Guinea Pigs. Investig. Ophthalmol. Vis. Sci..

[67] Harb E.N., Wildsoet C.F. (2019). Origins of Refractive Errors: Environmental and Genetic Factors. Annu. Rev. Vis. Sci..

[68] Flitcroft D.I. (2014). Emmetropisation and the Aetiology of Refractive Errors. Eye.

