# Supplementary material for: Axial Length to Corneal Radius of Curvature Ratio (AL/CR) and Refractive Errors in a Single Center Romanian Population
Source: Biomedicines. 2025 Nov 10;13(11):2742. doi: 10.3390/biomedicines13112742 (PMC12650697; doi:10.3390/biomedicines13112742)
Supplement: Supplementary file 1 [file biomedicines-13-02742-s001.zip › biomedicines-3935470-supplementary.pdf]

biomedicines

MDPI

Supplementary materials

Table S1. Baseline characteristics table summarizing key demographic and ocular parameters of the study population, primary stratification by refractive error (myopia, emmetropia, hyperopia), secondary by age group and sex.

Study group	Age		Gender	N	Mean	Std. Deviation	Std. Error Mean
			Male	2	-23.150	9.192	6.500
		SE (D)	Female	6	-42.317	278.312	113.620
			Male	2	113.500	91.924	65.000
		CH (mmHg)	Female	6	124.833	212.077	86.580
			Male	2	101.000	98.995	70.000
		CRF (mmHg)	Female	6	123.333	223.308	91.165
			Male	2	56.750	707.000	0.001
		CCT (mm)	Female	6	0.545	0.039	0.016
			Male	2	3.410	0.028	0.020
		ACD (mm)	Female	6	3.970	0.256	0.104
			Male	2	24.545	0.106	0.075
		AL (mm)	Female	6	24.635	1.674	0.684
			Male	2	7.820	0.014	0.010
		CR (mm)	Female	6	7.545	0.258	0.105
			Male	2	3.140	0.014	0.010
	6 - 10	AL/CR	Female	6	32.650	16.920	6.908
			Male	17	-36.200	169.808	41.184
		SE (D)	Female	14	-28.957	115.934	30.985
			Male	17	127.353	114.888	27.864
Myopia (M)	11 - 14	CH (mmHg)	Female	14	122.500	190.253	50.847

		CRF (mmHg)	Male	17	128.118	137.017		33.231
			Female	14	126.286	122.376		32.706
			Male	17	57.035	17.906		4.343
		CCT (mm)	Female	14	55.750	30.741		8.216
			Male	17	38.935	30.313		7.352
		ACD (mm)	Female	12	39.717	8.922		2.576
			Male	17	252.694	90.064		21.844
		AL (mm)	Female	14	245.293	117.795		31.482
			Male	17	77.841	26.576		6.446
		CR (mm)	Female	14	76.964	39.361		10.520
			Male	17	32.471	7.287		1.767
		AL/CR	Female	14	31.886	6.573		1.757
			Male	19	-24.358	150.975		34.636
		SE (D)	Female	18	-33.978	204.670		48.241
			Male	19	117.053	157.920		36.229
		CH (mmHg)	Female	18	111.389	160.115		37.740
		CRF (mmHg)	Male	19	125.737	260.146		59.682
			Female	18	112.500	172.840		40.739
			Male	19	58.068	46.878		10.755
		CCT (mm)	Female	18	53.272	41.366		9.750
			Male	18	39.650	33.806		7.968
		ACD (mm)	Female	18	37.339	34.062		8.029
			Male	19	250.721	90.827		20.837
		AL (mm)	Female	18	247.356	88.561		20.874
	15 - 18	CR (mm)	Male	19	78.816	21.869		5.017

			Female	18	76.783	25.063	5.907
			Male	19	31.816	11.182	2.565
		AL/CR	Female	18	32.233	9.016	2.125
			Male	21	-30.800	233.023	50.850
		SE (D)	Female	47	-28.909	218.211	31.829
			Male	21	110.429	116.601	25.444
		CH (mmHg)	Female	47	110.683	150.287	21.922
			Male	21	112.571	173.625	37.888
		CRF (mmHg)	Female	47	108.894	139.038	20.281
			Male	21	55.805	39.301	8.576
		CCT (mm)	Female	47	54.300	33.147	4.835
			Male	19	37.321	27.038	6.203
		ACD (mm)	Female	43	36.677	32.067	4.890
			Male	21	248.962	104.252	22.750
		AL (mm)	Female	47	242.651	128.391	18.728
			Male	21	77.957	24.305	5.304
		CR (mm)	Female	47	76.149	21.691	3.164
			Male	21	31.967	16.042	3.501
	>18	AL/CR	Female	47	31.864	13.890	2.026
			Male	4	2.225	18.500	9.250
		SE (D)	Female	5	2.000	36.187	16.183
			Male	4	135.750	47.170	23.585
		CH (mmHg)	Female	5	149.600	271.441	121.392
			Male	4	123.000	50.332	25.166
		CRF (mmHg)	Female	5	143.200	286.740	128.234
Emmetropia (E)	6 - 10	CCT (mm)	Male	4	57.350	3.317	1.658

			Female	5	57.360	42.606	19.054
			Male	4	37.050	20.469	10.235
		ACD (mm)	Female	5	34.340	7.603	3.400
			Male	4	231.125	37.464	18.732
		AL (mm)	Female	5	229.100	24.042	10.752
			Male	4	77.275	11.500	5.750
		CR (mm)	Female	5	77.740	10.807	4.833
			Male	4	29.900	816.000	408.000
		AL/CR	Female	5	29.460	2.510	1.122
			Male	10	-380.000	16.033	5.070
		SE (D)	Female	10	870.000	35.979	11.377
			Male	10	125.800	46.857	14.817
		CH (mmHg)	Female	10	126.900	231.490	73.204
			Male	10	128.900	119.764	37.873
		CRF (mmHg)	Female	10	129.000	259.315	82.003
			Male	10	58.800	41.061	12.985
		CCT (mm)	Female	10	55.900	46.068	14.568
			Male	10	37.670	14.384	4.549
		ACD (mm)	Female	8	37.913	5.249	1.856
			Male	10	247.640	89.729	28.375
		AL (mm)	Female	10	233.500	76.697	24.254
			Male	10	81.360	31.595	9.991
		CR (mm)	Female	10	77.400	30.478	9.638
			Male	10	30.450	3.240	1.025
	11 - 14	AL/CR	Female	10	30.180	2.936	929.000

			Male	5	-2.760	37.159	16.618
		SE (D)	Female	12	-1.658	34.888	10.071
			Male	5	127.200	295.753	132.265
		CH (mmHg)	Female	12	130.167	100.800	29.098
			Male	5	127.600	275.372	123.150
		CRF (mmHg)	Female	12	131.500	152.762	44.099
			Male	5	58.800	51.103	22.854
		CCT (mm)	Female	12	57.583	26.031	7.514
			Male	3	38.767	26.558	15.333
		ACD (mm)	Female	11	38.073	37.073	11.178
			Male	5	239.220	78.104	34.929
		AL (mm)	Female	12	231.383	103.907	29.995
			Male	5	78.080	22.005	9.841
		CR (mm)	Female	12	76.283	31.634	9.132
			Male	5	30.640	7.092	3.172
	15 - 18	AL/CR	Female	12	30.333	5.245	1.514
			Male	18	694.000	30.532	7.196
		SE (D)	Female	28	671.000	30.941	5.847
			Male	18	108.833	221.817	52.283
		CH (mmHg)	Female	28	117.964	162.264	30.665
			Male	18	114.000	228.756	53.918
		CRF (mmHg)	Female	28	118.607	177.187	33.485
			Male	18	55.772	35.516	8.371
		CCT (mm)	Female	28	56.043	38.153	7.210
	>18	ACD (mm)	Male	18	35.917	24.457	5.765

			Female	28	34.589	24.045	4.544
			Male	18	236.567	51.965	12.248
		AL (mm)	Female	28	234.664	55.452	10.480
			Male	18	77.439	15.309	3.608
		CR (mm)	Female	28	77.543	22.521	4.256
			Male	18	30.550	4.162	981.000
		AL/CR	Female	28	30.264	5.201	983.000
			Male	6	22.317	190.964	77.961
		SE (D)	Female	11	12.982	31.597	9.527
			Male	6	129.167	211.983	86.542
		CH (mmHg)	Female	11	129.545	180.796	54.512
			Male	6	125.000	213.354	87.101
		CRF (mmHg)	Female	11	131.455	205.054	61.826
			Male	6	56.600	21.279	8.687
		CCT (mm)	Female	11	56.664	35.449	10.688
			Male	6	36.217	37.091	15.142
		ACD (mm)	Female	9	35.989	23.646	7.882
			Male	6	228.100	112.574	45.958
		AL (mm)	Female	11	227.845	53.208	16.043
			Male	6	79.000	17.297	7.062
		CR (mm)	Female	11	77.409	19.154	5.775
			Male	6	28.883	10.439	4.262
	6 - 10	AL/CR	Female	11	29.436	4.739	1.429
			Male	5	11.780	48.792	21.821
		SE (D)	Female	5	33.780	184.467	82.496
Hyperopia (H)	11 - 14	CH (mmHg)	Male	5	121.000	327.261	146.356

			Female	5	142.200	216.032	96.613
		CRF (mmHg)	Male	5	116.200	320.811	143.471
			Female	5	134.800	192.016	85.872
		CCT (mm)	Male	5	54.560	9.990	4.468
			Female	5	55.600	45.951	20.550
		ACD (mm)	Male	5	37.880	12.834	5.739
			Female	5	37.000	17.436	7.797
		AL (mm)	Male	5	224.700	110.917	49.603
			Female	5	223.000	24.779	11.082
		CR (mm)	Male	5	74.760	49.303	22.049
			Female	5	77.460	30.022	13.426
		AL/CR	Male	5	30.060	5.683	2.542
			Female	5	28.800	8.916	3.987
	15 - 18	SE (D)	Male	1	7.500	.	.
			Female	1	6.300	.	.
		CH (mmHg)	Male	1	169.000	.	.
			Female	1	128.000	.	.
		CRF (mmHg)	Male	1	168.000	.	.
			Female	1	143.000	.	.
		CCT (mm)	Male	1	63.200	.	.
			Female	1	63.200	.	.
		ACD (mm)	Male	1	35.700	.	.
			Female	1	34.500	.	.
		AL (mm)	Male	1	227.800	.	.
			Female	1	232.000	.	.

			Male	1	77.800	.	.
		CR (mm)	Female	1	77.400	.	.
			Male	1	29.300	.	.
		AL/CR	Female	1	30.000	.	.
			Male	4	21.900	304.053	152.026
		SE (D)	Female	14	20.193	156.475	41.820
			Male	4	127.000	107.393	53.697
		CH (mmHg)	Female	14	117.857	200.724	53.646
			Male	4	118.500	221.284	110.642
		CRF (mmHg)	Female	14	119.429	229.940	61.454
			Male	4	56.575	22.559	11.280
		CCT (mm)	Female	14	56.779	38.375	10.256
			Male	3	36.000	42.438	24.502
		ACD (mm)	Female	14	30.864	30.173	8.064
			Male	4	231.250	109.537	54.768
		AL (mm)	Female	14	225.229	30.009	8.020
			Male	4	76.875	9.359	4.679
		CR (mm)	Female	14	76.479	18.664	4.988
			Male	4	30.075	15.218	7.609
	>18	AL/CR	Female	14	29.464	7.581	2.026
